# *GALNT14* Genotype Predicts Postoperative Outcome of Stage III Colorectal Cancer With Oxaliplatin as Adjuvant Chemotherapy

**DOI:** 10.1097/MD.0000000000003487

**Published:** 2016-04-29

**Authors:** Wey-Ran Lin, Jy-Ming Chiang, Kung-Hao Liang, Siew-Na Lim, Ming-Wei Lai, Yung-Kuan Tsou, Tzu-Yun Hsieh, Chih-Kai Hsu, Chau-Ting Yeh

**Affiliations:** From the Department of Gastroenterology and Hepatology (W-RL, Y-KT, C-TY); Liver Research Center (W-RL, K-HL, M-WL, Y-KT, C-TY), Linkou Chang Gung Memorial Hospital; Chang Gung University College of Medicine (W-RL, J-MC, S-NL, M-WL, Y-KT, T-YH, C-KH, C-TY); Department of Colorectal Surgery (J-MC); Department of Neurology (S-NL), Linkou Chang Gung Memorial Hospital; and Department of Pediatrics (M-WL), Chang Gung Children's Hospital, Taoyuan, Taiwan.

## Abstract

Adjuvant oxaliplatin-based chemotherapy is widely used for stage III colorectal cancer (CRC) after curative surgery. CRC is a molecularly heterogeneous disease, and our current knowledge of therapeutic response-related genetic factors remains limited. *N*-acetylgalactosaminyltransferase 14 (*GALNT14*)-rs9679162 genotype is a prognostic predictor for chemotherapy response in advanced hepatocellular carcinoma. Here, we investigated whether this genotype was related to the therapeutic outcome of stage III CRC.

A cohort of 300 stage III CRC patients receiving curative resection followed by oxaliplatin-based chemotherapy was retrospectively recruited. *GALNT14* genotypes and the clinicopathological factors were correlated with posttherapeutic prognosis.

Of these patients, 18% patients had *GALNT14*-rs9679162 “TT” and 82% had the “GT” + “GG” genotypes. The analysis showed that the “TT” genotype was associated with unfavorable overall survival (OS, *P* = 0.009) but not with recurrence-free survival (RFS, *P* = 0.700). The subgroup analysis showed that the “TT” genotype was associated with unfavorable OS in the following subgroups: age ≤65 years, men, left side CRC, N2 stage, carcinoembryonic antigen >5 ng/mL, and mucinous histology (*P* = 0.012, 0.011, 0.009, 0.025, 0.013, and 0.007, respectively). Within the latter 2 subgroups, the “TT” genotype was the only independent predictor for OS. Finally, the “TT” genotype was associated with the T4 tumor stage (*P* = 0.017) and in patients with T4 tumors, the “TT” genotype was the only independent predictor for unfavorable RFS (*P* = 0.007).

*GALNT14* “TT” genotype was associated with unfavorable OS in stage III CRC patients receiving curative surgery and adjuvant oxaliplatin-based chemotherapy.

## Abstract 2

Supplemental Digital Content is available in the text

## INTRODUCTION

Colorectal cancer (CRC) is the third most common cause of cancer-related deaths worldwide. Among all newly diagnosed CRCs, stage III CRC constitutes the greatest proportion.^1^ Oxaliplatin-based systemic adjuvant chemotherapy has been shown to be beneficial for stage III CRC patients in a number of trials and therefore has been suggested as a standard treatment for patients with stage III diseases.^2–4^ The benefit of adjuvant therapy includes an ∼30% reduction in risk of disease recurrence and a 22% to 32% reduction of mortality. It has been found that patients with stage III CRC are not a single aggregate with homogeneous risk but rather consisted of variable subsets of patients with different outcomes. The 5-year survival rate varies from 73% in stage IIIA (T1–2N1) to only 28% in stage IIIC CRC (N2).^5,6^ Moreover, CRC is a molecularly heterogeneous disease, and genetic analysis of the cancerous tissues revealed candidate prognostic markers such as 18q loss of heterozygosity, microsatellite instability, and large deletions in heat shock protein 110.^7–10^ Despite the success of these elegant studies, clinical applications of these markers are greatly limited by the accessibility to well-established molecular laboratories and the availability of standardized clinical assays. Therefore, there remain unmet needs for easily accessible genetic makers that can predict prognosis of CRC.

By the use of genome-wide association method followed by prospective validation, it was found that the genotype of polypeptide *N*-acetylgalactosaminyltransferase 14 (*GALNT14*) could be used as a prognostic predictor for chemotherapy for hepatocellular carcinoma.^11^ A leading single nucleotide polymorphism (SNP) marker, rs9679162, was capable of predicting the time-to-tumor progression, overall survival (OS), and responses to chemotherapy. In patients with advanced hepatocellular carcinoma receiving palliative chemotherapy, the “TT” genotype predicted a favorable treatment outcome.^12,13^ The product of *GALNT14* gene was a catalytic enzyme that catalyzed *O*-glycosylation of many proteins including the death receptor (DR)-4 and -5. Because the *O*-glycosylation of DR 4 of 5 increased their sensitivity to apoptotic signals, this genotype association of chemotherapy sensitivities might not be restricted to hepatocellular carcinoma. In CRC cells, it has been demonstrated that the increase of DR-5 enhances cytotoxic effects of 5-fluorouracil and oxaliplatin.^14^ Here, we hypothesized that the *GALNT14* genotype might also affect the outcome of stage III CRC patients and examined the clinicopathological parameters in relationship to *GALNT14* genotype and the prognosis predictive values of these factors.

## METHODS

### Patients

This study was conducted under the approval of the institutional review board of Chang Gung Memorial Hospital, Taiwan. Surgical samples of 300 stage III CRC patients resected between years 2003 and 2013 were retrieved from the hospital's tissue bank without other particular selection criteria. The stage was based on tumor-node-metastasis classification. CRCs with regional lymph nodes involvement (N1, metastasis in 1 to 3 regional lymph nodes; N2, metastasis in ≥4 regional lymph nodes), and tumor invasion (T1, submucosa; T2, muscularis propria; T3, pericolorectal tissues; T4, direct invasion or adherent to other organ or structures) but without distant metastasis (M0) were classified as stage III. All the patients received oxaliplatin-based adjuvant chemotherapies after curative resection. The clinicopathological data were collected including age, sex, carcinoembryonic antigen (CEA), tumor location, tumor size, tumor surface area, tumor-free margin, tumor invasion, regional lymph node involvement, tumor differentiation, histology type, and chemotherapy regimen used.

### Chemotherapy Regimens

Two oxaliplatin-based regimens, modified Folinic acid, Fluorouracil, Oxaliplatin 6 (mFOLFOX6) and capecitabine plus oxaliplatin (XELOX), were given to patients after curative resection. The mFOLFOX6 was given as the following: oxaliplatin (dose 85 mg/m^2^) and leucovorin (400 mg/m^2^) were administered continuously over 2 hours via intravenous route on day 1. The 5-fluorouracil was given intravenous bolus at the dose of 400 mg/m^2^ on day 1, followed by 1200 mg/m^2^/day for 2 days (total 2400 mg/m^2^ over 46–48 hours) with continuous intravenous infusion. The mFOLFOX6 regimen was repeated every 2 weeks for 12 cycles. The XELOX protocol was given as the following: oxaliplatin (130 mg/m^2^) was continuously administered via intravenous route for 2 hours on day 1. Capecitabine (1700–2000 mg/m^2^) was given via oral route on day 1 to day 14. The XELOX regimen was repeated every 3 weeks for 8 cycles.

### *GALNT14* Genotyping

Genotyping of *GALNT14* was performed as described in our previous publications.^12,13^ Briefly, tissue DNA in the paraffin blocks of CRC was extracted and purified. Two primers, 5’-TCACGAGGCCAACATTCTAG-3’ and 5’-TTAGATTCTGCATGGCTCAC-3’, were synthesized, which flanked a 172-bp intronic region of *GALNT14* gene containing rs9679162. The SNP genotype was determined by direct sequencing after polymerase chain reaction amplification.

### Statistical Analysis

The details of statistical methods performed in this study have been described in our previous publication.^12^

## RESULTS

### Basic Clinical Data of the Stage III CRC Patients Included

A total of 300 stage III CRC patients, who received radical resection and oxaliplatin-based adjuvant chemotherapy, were included. The basic clinical data were listed in Table 1. The *GALNT14* genotype was determined based on the sequence of DNA obtained from tissue blocks. Of them, 54 (18%) patients were “TT” type and 246 (82%) were “non-TT” type (“GG” + “GT”). The proportion of “TT” type was significantly lower than those in the HapMap Chinese Han Beijing (30.15%) and Metropolitan Denver (28.44%) cohorts (χ^2^ test *P* = 0.0044 and 0.0214) respectively.^15^ Most clinicopathological factors showed no significant association to *GALNT14* genotype (Table 1). Only 1 exception, the T4 tumor stage, which was significantly more prevalent in patients with the “TT” genotype compared with those with “non-TT” genotype (35.2% vs 19.1%, *P* = 0.017, Table 1).

**TABLE 1:**
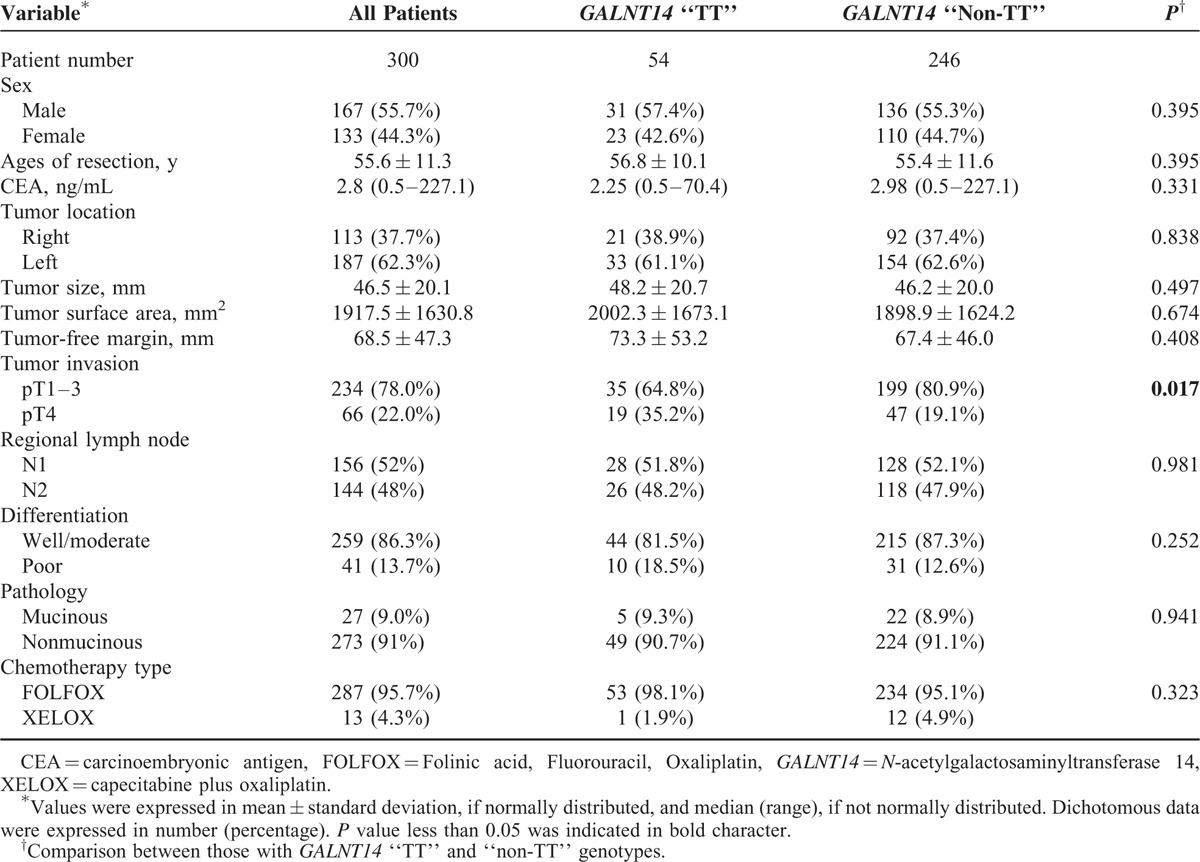
Basic Clinicopathological Factors of Patients Included

### *GALNT14* Genotype in Association With OS

**FIGURE 1:**
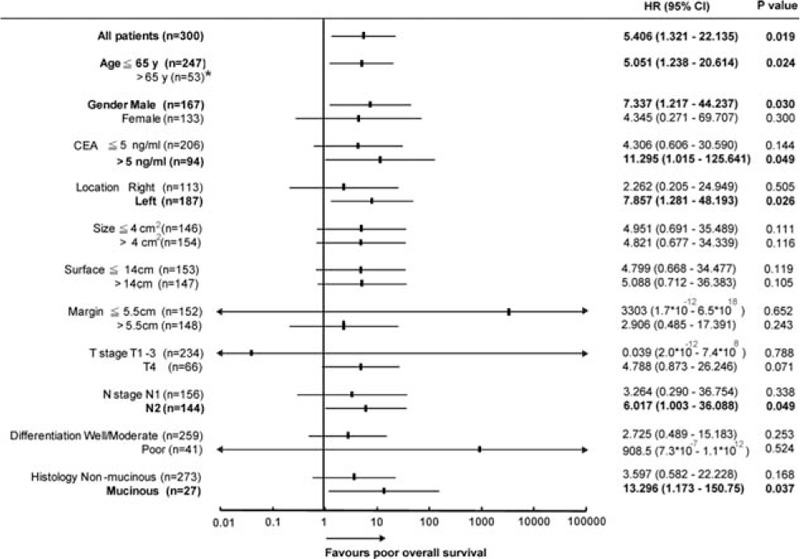
Forest plot of HRs for the impact of *GALNT14* “TT” genotype in terms of OS by clinicopathological parameters. The subgroup-specific odds ratios (95% CI) were denoted by black boxes (black lines). Bold text indicate a statistically significant difference with *P* < 0.05. ^∗^No event was observed in patients with ages >65 years. CI = confidence interval, *GALNT14* = *N*-acetylgalactosaminyltransferase 14, HR = hazard ratio, OS = overall survival.

**FIGURE 2:**
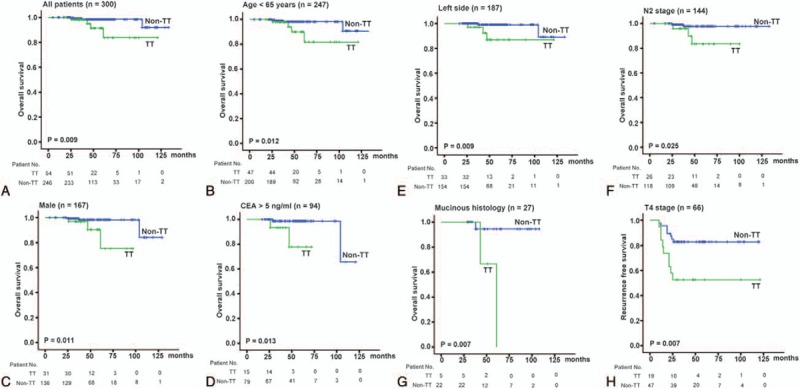
Kaplan–Meier survival curves with log-rank test stratified by *GALNT14* “TT” and “non-TT” genotypes. (A) The OS of stage III CRC patients (n = 300, *P* = 0.009). (B) The OS of patients with ages ≤65 years (n = 247, *P* = 0.012). (C) The OS of male patients (n = 167, *P* = 0.011). (D) The OS of patients with CEA >5 ng/mL (n = 94, *P* = 0.013). (E) The OS of patients with left-side CRC (n = 187, *P* = 0.009). (F) The OS of patients with N2 stage (n = 144, *P* = 0.025). (G) The OS of patients with mucinous CRC (n = 27, *P* = 0.007). (H) The RFS of patients with T4 stage (n = 66, *P* = 0.007). CEA = carcinoembryonic antigen, CRC = colorectal cancer, *GALNT14* = *N*-acetylgalactosaminyltransferase 14, OS = overall survival, RFS = recurrence-free survival.

To study whether *GALNT14* genotype was associated with prognosis, we retrieved the longitudinal data of recurrence-free survival (RFS) and OS. The median posttherapeutic follow-up period was 47 (5–133) months. Cox proportional hazard method and Kaplan–Meier plot with log-rank analysis showed there was no significance between the “TT” genotype and RFS (Cox *P* = 0.701, log-rank *P* = 0.700). However, the patients with “TT” genotype had a significantly shorter OS compared with patients with “non-TT” genotype (Figures 1 and 2A, Cox *P* = 0.019, log-rank *P* = 0.009).

Subsequently, we examined the predictive role of *GALNT14* “TT” genotype in various clinical subgroups using Cox proportional hazard method (Figure 1). These data were further examined using the Kaplan–Meier plot with log-rank analysis. The “TT” and “non-TT” patients had distinguishable survival curves not only in all stage III CRC patients included but also in the following subgroups: age ≤65 years (Figure 2B, *P* = 0.012), men (Figure 2C, *P* = 0.011), CEA >5 ng/mL (Figure 2D, *P* = 0.013), left CRC (Figure 2E, *P* = 0.009), N2 stage (Figure 2F, *P* = 0.025), and mucinous histology (Figure 2G, *P* = 0.007).

### Other Clinicopathological Predictors for RFS and OS

To identify other clinicopathological predictors for RFS and OS, univariate followed by multivariate Cox proportional hazard analysis was performed (Table 2). The CEA level (*P* = 0.030), tumor size (*P* = 0.012), surface area (*P* = 0.022), and N stage (*P* = 0.014) were associated with RFS by univariate analysis. After adjusting for the confounding factors, multivariate analysis showed that CEA level (*P* = 0.027) and N stage (*P* = 0.004) were independent predictors for RFS.

**TABLE 2:**
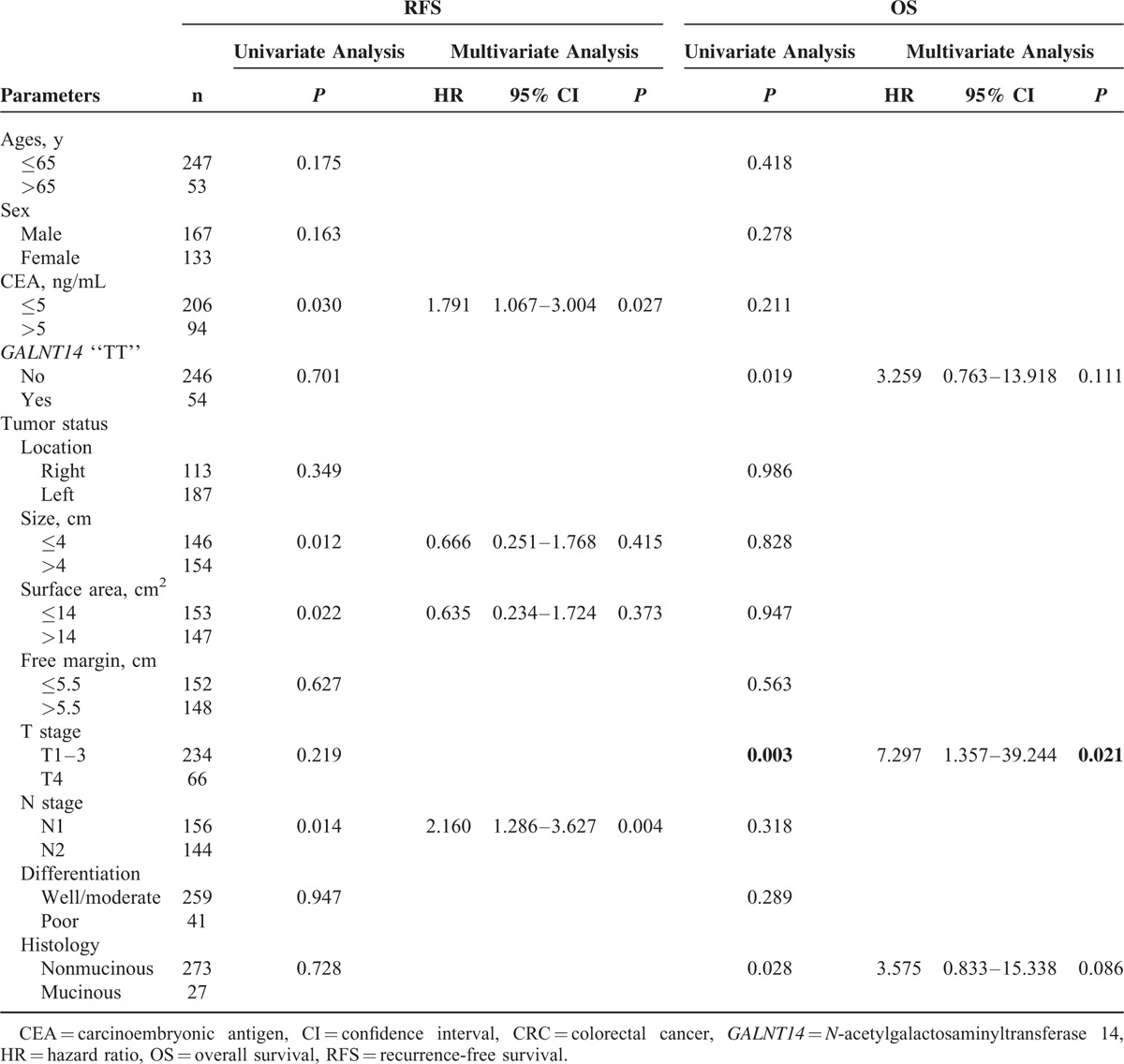
Analysis of Factors That Influenced RFS and OS Using Data of All Stage III CRC Patients (n = 300)

For OS, the *GALNT14* “TT” genotype (*P* = 0.019), the T4 stage (*P* = 0.003), and the mucinous histology (*P* = 0.028) were associated with poor prognosis by univariate Cox proportional models. Multivariate analysis revealed that only T4 stage (*P* = 0.021) was the independent predictor for OS.

### *GALNT14* “TT” Genotype as an Independent Outcome Predictor in Clinical Subgroups

Because *GALNT14* genotypes were associated with both T4 stage (Table 1) and OS (Figures 1 and 2), we subsequently examined the predictive value of *GALNT14* “TT” genotype in the T4 stage patient subgroup (n = 66, Table 3). The result showed that “TT” genotype was the only independent predictor for RFS in this subgroup (Figure 2H, Cox *P* = 0.024, log-rank *P* = 0.007). For OS, the mucinous histology was the only unfavorable predictor (Cox *P* = 0.040, log-rank *P* = 0.021).

**TABLE 3:**
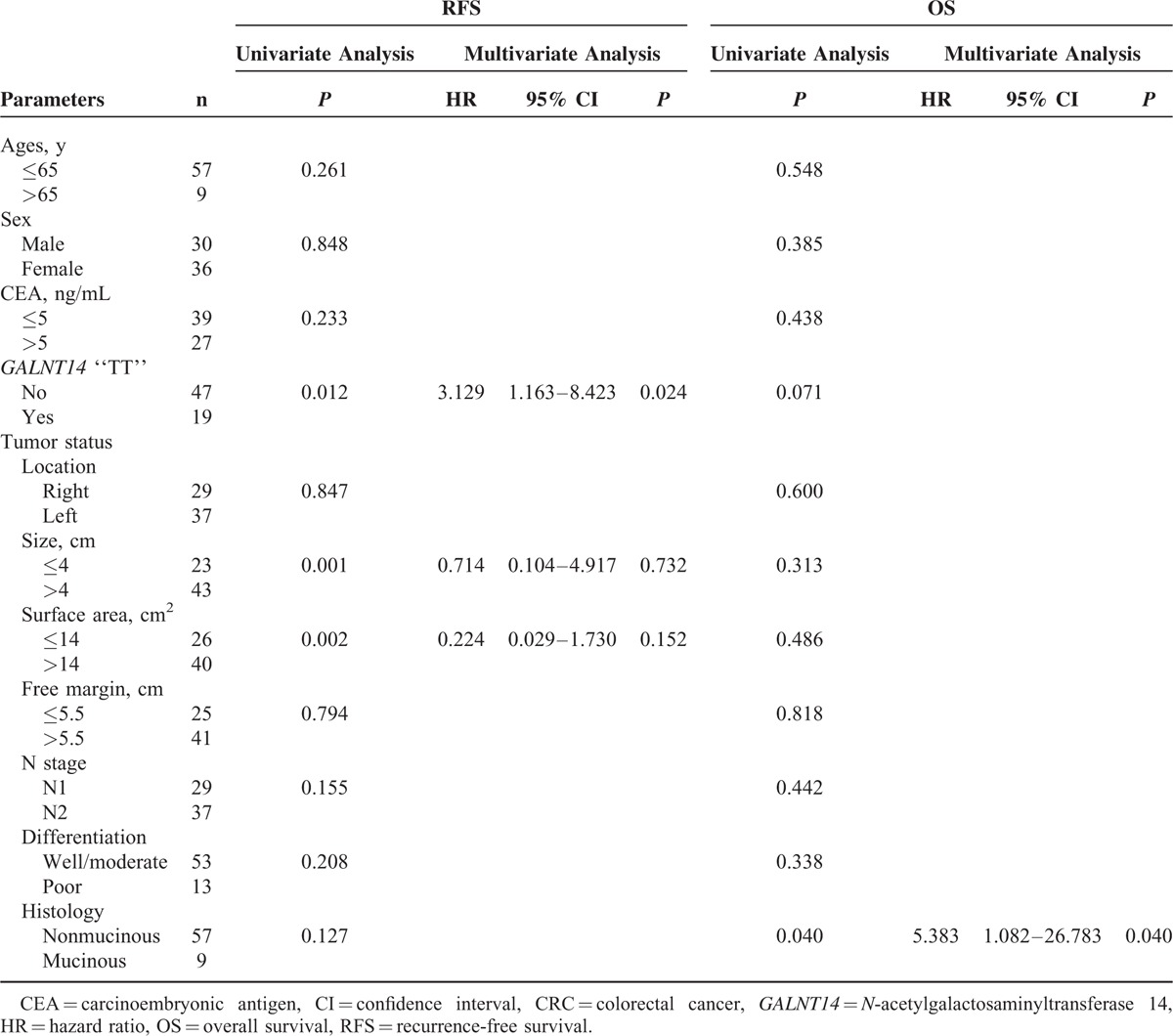
Analysis of Factors That Influenced RFS and OS Using Data of Patients With T4 Stage (n = 66)

In addition to T4 stage subgroup, we also analyzed the prognosis predictive value of *GALNT14* genotype in other clinical subgroups associated with poor OS (Figure 1). It was found that in patients with CEA >5 ng/mL (n = 94, supplementary Table 1), “TT” genotype was the only unfavorable factor for OS (Cox *P* = 0.049). In patients with mucinous histology (n = 27, supplementary Table 2), the “TT” genotype was the only unfavorable predictor for OS (Cox *P* = 0.037). Other analysis for the predictive value of *GALNT14* genotype in clinical subgroups was given in supplementary Tables 3–6.

## DISCUSSION

At this time, routine adjuvant chemotherapeutic treatment is recommended for patients with high-risk stage II and stage III CRC. It has been documented that oxaliplatin-based adjuvant chemotherapy confers survival advantage in stage III CRC patients. However, no pretreatment prognostic molecular biomarkers for stage III CRC receiving oxaliplatin-based adjuvant chemotherapy regimen has been established until now. The present study demonstrated that the *GALNT14* “TT” genotype was positively associated with T4 stage, a well-known poor prognostic predictor. Further analysis showed that the “TT” genotype was associated with poor OS in all included patients and several clinical subgroups, including age ≤65 years, men, CEA >5 ng/mL, left-side CRC, N2 stage, and mucinous histology.

In addition to *GALNT14* “TT” genotype, several poor prognostic subgroups were identified including CEA >5 ng/mL, N2 stage, T4 stage, and mucinous histology. This is consistent with previous studies from other groups.^1,16,17^ To identify useful prognostic predictors within these subgroups, we further analyzed the RFS and OS with respect to the clinicopathological parameters and *GALNT14* genotype. Intriguingly, *GALNT14* was again identified as a valuable prognostic predictor in these subgroups. For patients in T4 stage, the “TT” genotype was significantly associated with poor RFS. For patients with CEA >5 ng/mL or mucinous histology, the “TT” genotype was the only predictor associated with poor OS.

Like many other cancers, development and progression of CRC are affected by multiple factors. Most research efforts have been focused on the underlying molecular mechanisms of CRC carcinogenesis, and a variety of candidate genetic markers with predictive value of outcome have been discovered by studying the cancerous tissues (e.g., *KRAS* expression, levels of DNA mismatch repair, *18q* deletion, and *p53* expression).^7–9,18,19^ However, personal genetic background might contribute greatly to this disease. Our study showed that patients with *GALNT14* “TT” genotype had a higher rate to develop tumor invasion (T4 stage) and thus have an unfavorable OS. Furthermore, the “TT” genotype was associated with poor RFS in T4 stage patients. Thus, *GALNT14* “TT” genotype has 2 major clinicopathological implications. First, tumors with *GALNT14* “TT” genotype have more aggressive cancer invasion property during oncogenesis. Second, tumors with “TT” genotype may have decreased drug sensitivity to oxaliplatin, which is reflected from its association with poor RFS in T4 stage patients. Therefore, other more potent cytotoxic agents should be considered in patients with *GALNT14* “TT” genotype.

Oxaliplatin is a platin analogue that has been widely used in treating a variety of solid tumors, especially in CRCs. It induces apoptosis and cell death through the inhibition of DNA replication by forming intrastrand cross-link DNA adducts.^20^ Several factors may affect the therapeutic outcome of oxaliplatin-based chemotherapy including genetic polymorphism. It has been demonstrated that metastatic CRC with the SNP located within excision repair cross-complementing rodent repair deficiency complementation group1 (*ERCC1*) codon 118C/T or T/T genotype was associated with worse survival,^21–23^ whereas other groups showed that metastatic CRC patients with the same genotype had better response or survival compared with C/C patients.^24,25^ Although the results on the predictive value of *ERCC1* codon 118 polymorphism have been inconsistent, these studies raise the possibility that genetic SNP might play a role in therapeutic response. Our current study of *GALNT14* genotype supported this view. Moreover, the gene product of *GALNT14* is an enzyme that catalyzes *O*-glycosylation of many proteins including the DR-4 and -5. It has been reported that the cytotoxic effects of oxaliplatin in CRC can be enhanced by tenovin-6 through upregulating DR-5,^14^ suggesting that modulation of DR-mediated signaling may affect the effects of oxaliplatin-based chemotherapy. In this study, 1 convenient explanation for the *GALNT14* genotypes–associated therapeutic outcomes is the differential intensities of DR-mediated apoptosis signaling between the “TT” and “non-TT” genotypes. However, because *O*-glycosylation is a body-wise process, it is unclear whether there are other unrecognized important glycoproteins involved. Moreover, several genetic alternations in colorectal cancerous tissues, such as 18q loss of heterozygosity, microsatellite instability, and large deletions in heat shock protein 110, have been proposed as candidate prognostic markers, but the association between *GALNT14* genotypes and these genetic makers is unclear. Further studies to clarify these issues are needed.

This study had several limitations, including its retrospective nature, limited case number, and restriction of the sample to a Chinese population from a single medical center. Our eligibility criteria confined the treatment to oxaliplatin-based chemotherapies (mFOLFOX6 and XELOX) for interpretative clarity. Therefore, it may not pertain to other regimens not containing oxaliplatin. The decision of which oxaliplatin-based regimens to be used was not only dependent on clinical criteria but also on nonclinical considerations such as doctors’ decision and patients’ willingness. The age of patients in this cohort study was relatively young compared with other CRC cohort studies. This was because elderly CRC patients were more often willing to have chemotherapy via oral route than intravenous route. Despite all these drawbacks, our study results are considered useful in routine clinical practice.

In conclusion, *GALNT14* “TT” genotype was correlated with T4 stage and associated with poor OS in stage III CRC patients receiving curative resection and adjuvant oxaliplatin-based chemotherapy. It was further associated with poor treatment outcome in subgroups of T4 stage, CEA >5 ng/mL, or mucinous histology. Taken together, *GALNT14* genotype is a valuable prognostic predictor in advanced CRC.

## Supplementary Material

Supplemental Digital Content
