# Supplementary material for: GALNT14 Genotype Predicts Postoperative Outcome of Stage III Colorectal Cancer With Oxaliplatin as Adjuvant Chemotherapy
Source: Medicine (Baltimore). 2016 Apr 29;95(17):e3487. doi: 10.1097/MD.0000000000003487 (PMC4998711; doi:10.1097/MD.0000000000003487)
Supplement: Supplemental Digital Content [file medi-95-e3487-s001.doc]

Supplementary Table 1. Analysis of factors that influenced recurrence free survival and overall survival using data of patients with CEA >5 ng/ml (n=94)

|  | | | | **Recurrence Free Survival** | | | | |  | **Overall Survival** | | | | | |
| --- | --- | --- | --- | --- | --- | --- | --- | --- | --- | --- | --- | --- | --- | --- | --- |
|  | |  | | **Univariate Analysis** |  | **Multivariate Analysis** | | |  | **Univariate Analysis** |  | **Multivariate Analysis** | | | |
| **Parameters** | | **n** | | **P value** |  | **HR** | **95% CI** | **P value** |  | **P value** |  | **HR** | | **95% CI** | **P value** |
| Ages (years) | ≤ 65 | | 71 | 0.622 |  |  |  |  |  | 0.473 |  |  | |  |  |
|  | > 65 | | 23 |  |  |  |  |  |  |  |  |  | |  |  |
| Gender | Male | | 52 | 0.860 |  |  |  |  |  | 0.572 |  |  | |  |  |
|  | Female | | 42 |  |  |  |  |  |  |  |  |  | |  |  |
| GALNT14 “TT” | No | | 79 | 0.491 |  |  |  |  |  | **0.049** |  | 11.295 | 1.015-125.641 | | **0.049** |
|  | Yes | | 15 |  |  |  |  |  |  |  |  |  |  | |  |
| **Tumor status** |  | |  |  |  |  |  |  |  |  |  |  |  | |  |
| Location | Right | | 37 | 0.131 |  |  |  |  |  | 0.802 |  |  |  | |  |
|  | Left | | 57 |  |  |  |  |  |  |  |  |  |  | |  |
| Size (cm) | ≤4 | | 44 | **0.017** |  | 0.594 | 0.139-2.534 | 0.482 |  | 0.463 |  |  |  | |  |
|  | >4 | | 50 |  |  |  |  |  |  |  |  |  |  | |  |
| Surface area (cm^2^) | ≤14 | | 43 | **0.016** |  | 0.570 | 0.134-2.430 | 0.447 |  | 0.483 |  |  |  | |  |
|  | >14 | | 51 |  |  |  |  |  |  |  |  |  |  | |  |
| Free margin (cm) | ≤ 5.5 | | 49 | 0.491 |  |  |  |  |  | 0.465 |  |  |  | |  |
|  | > 5.5 | | 45 |  |  |  |  |  |  |  |  |  |  | |  |
| T stage | T1-3 | | 67 | 0.367 |  |  |  |  |  | 0.365 |  |  |  | |  |
|  | T4 | | 27 |  |  |  |  |  |  |  |  |  |  | |  |
| N stage | N1 | | 50 | 0.065 |  |  |  |  |  | 0.400 |  |  |  | |  |
|  | N2 | | 44 |  |  |  |  |  |  |  |  |  |  | |  |
| Differentiation | Well/moderate | | 82 | 0.826 |  |  |  |  |  | 0.281 |  |  |  | |  |
|  | Poor | | 12 |  |  |  |  |  |  |  |  |  |  | |  |
| Pathology | Non-mucinous | | 87 | 0.955 |  |  |  |  |  | 0.745 |  |  |  | |  |
|  | Mucinous | | 7 |  |  |  |  |  |  |  |  |  |  | |  |

Abbreviations: CEA, carcinoembryonic antigen; CI, confidence interval; *GALNT14*, N-acetylgalactosaminyltransferase 14; HR, hazard ratio

Supplementary Table 2. Analysis of factors that influenced recurrence free survival and overall survival using data of patients with mucinous histology (n=27)

|  | | | | **Recurrence Free Survival** | | | | |  | **Overall Survival** | | | | | |
| --- | --- | --- | --- | --- | --- | --- | --- | --- | --- | --- | --- | --- | --- | --- | --- |
|  | |  | | **Univariate Analysis** |  | **Multivariate Analysis** | | |  | **Univariate Analysis** |  | **Multivariate Analysis** | | | |
| **Parameters** | | **n** | | **P value** |  | **HR** | **95% CI** | **P value** |  | **P value** |  | **HR** | | **95% CI** | **P value** |
| Ages (years) | ≤ 65 | | 22 | 0.486 |  |  |  |  |  | 0.591 |  |  | |  |  |
|  | > 65 | | 5 |  |  |  |  |  |  |  |  |  | |  |  |
| Gender | Male | | 16 | 0.406 |  |  |  |  |  | 0.900 |  |  | |  |  |
|  | Female | | 11 |  |  |  |  |  |  |  |  |  | |  |  |
| CEA (ng/ml) | ≤ 5 | | 20 | 0.409 |  |  |  |  |  | 0.591 |  |  | |  |  |
|  | > 5 | | 7 |  |  |  |  |  |  |  |  |  | |  |  |
| GALNT14 “TT” | No | | 22 | **0.011** |  | 6.341 | 0.834 – 48.228 | 0.074 |  | **0.037** |  | 13.296 | 1.173-150.746 | | **0.037** |
|  | Yes | | 5 |  |  |  |  |  |  |  |  |  |  | |  |
| **Tumor status** |  | |  |  |  |  |  |  |  |  |  |  |  | |  |
| Location | Right | | 17 | 0.254 |  |  |  |  |  | 0.208 |  |  |  | |  |
|  | Left | | 10 |  |  |  |  |  |  |  |  |  |  | |  |
| Size (cm) | ≤4 | | 7 | 0.681 |  |  |  |  |  | 0.752 |  |  |  | |  |
|  | >4 | | 20 |  |  |  |  |  |  |  |  |  |  | |  |
| Surface area (cm^2^) | ≤14 | | 7 | 0.681 |  |  |  |  |  | 0.752 |  |  |  | |  |
|  | >14 | | 20 |  |  |  |  |  |  |  |  |  |  | |  |
| Free margin (cm) | ≤ 5.5 | | 7 | 0.406 |  |  |  |  |  | 0.529 |  |  |  | |  |
|  | > 5.5 | | 20 |  |  |  |  |  |  |  |  |  |  | |  |
| T stage | T1-3 | | 18 | **0.035** |  | 6.843 | 0.660 – 70.968 | 0.107 |  | 0.348 |  |  |  | |  |
|  | T4 | | 9 |  |  |  |  |  |  |  |  |  |  | |  |
| N stage | N1 | | 16 | 0.074 |  |  |  |  |  | 0.252 |  |  |  | |  |
|  | N2 | | 11 |  |  |  |  |  |  |  |  |  |  | |  |
| Differentiation | Well/moderate | | 7 | 0.514 |  |  |  |  |  | 0.268 |  |  |  | |  |
|  | Poor | | 20 |  |  |  |  |  |  |  |  |  |  | |  |

Abbreviations: CEA, carcinoembryonic antigen; CI, confidence interval; CRC, colorectal cancer; *GALNT14*, N-acetylgalactosaminyltransferase 14; HR, hazard ratio

Supplementary Table 3. Analysis of factors that influenced recurrence free survival and overall survival using data of patients with N2 stage (n=144)

|  | | | | **Recurrence Free Survival** | | | | |  | **Overall Survival** | | | | | |
| --- | --- | --- | --- | --- | --- | --- | --- | --- | --- | --- | --- | --- | --- | --- | --- |
|  | |  | | **Univariate Analysis** |  | **Multivariate Analysis** | | |  | **Univariate Analysis** |  | **Multivariate Analysis** | | | |
| **Parameters** | | **n** | | **P value** |  | **HR** | **95% CI** | **P value** |  | **P value** |  | **HR** | | **95% CI** | **P value** |
| Ages (years) | ≤ 65 | | 118 | 0.091 |  |  |  |  |  | 0.502 |  |  | |  |  |
|  | > 65 | | 26 |  |  |  |  |  |  |  |  |  | |  |  |
| Gender | Male | | 77 | 0.665 |  |  |  |  |  | 0.303 |  |  | |  |  |
|  | Female | | 67 |  |  |  |  |  |  |  |  |  | |  |  |
| CEA (ng/ml) | ≤ 5 | | 100 | 0.037 |  | 1.988 | 1.042 - 3.793 | 0.037 |  | 0.593 |  |  | |  |  |
|  | > 5 | | 44 |  |  |  |  |  |  |  |  |  | |  |  |
| GALNT14 “TT” | No | | 118 | 0.932 |  |  |  |  |  | **0.049** |  | 3.462 | 0.527 – 22.731 | | 0.196 |
|  | Yes | | 26 |  |  |  |  |  |  |  |  |  |  | |  |
| **Tumor status** |  | |  |  |  |  |  |  |  |  |  |  |  | |  |
| Location | Right | | 45 | 0.799 |  |  |  |  |  | 0.661 |  |  |  | |  |
|  | Left | | 99 |  |  |  |  |  |  |  |  |  |  | |  |
| Size (cm) | ≤4 | | 66 | 0.074 |  |  |  |  |  | 0.362 |  |  |  | |  |
|  | >4 | | 78 |  |  |  |  |  |  |  |  |  |  | |  |
| Surface area (cm^2^) | ≤14 | | 69 | 0.152 |  |  |  |  |  | 0.408 |  |  |  | |  |
|  | >14 | | 75 |  |  |  |  |  |  |  |  |  |  | |  |
| Free margin (cm) | ≤ 5.5 | | 79 | 0.069 |  |  |  |  |  | 0.462 |  |  |  | |  |
|  | > 5.5 | | 65 |  |  |  |  |  |  |  |  |  |  | |  |
| T stage | T1-3 | | 107 | 0.256 |  |  |  |  |  | **0.022** |  | 7.185 | 0.696 – 74.157 | | 0.098 |
|  | T4 | | 37 |  |  |  |  |  |  |  |  |  |  | |  |
| Differentiation | Well/moderate | | 123 | 0.691 |  |  |  |  |  | 0.097 |  |  |  | |  |
|  | Poor | | 21 |  |  |  |  |  |  |  |  |  |  | |  |
| Pathology | Non-mucinous | | 133 | 0.425 |  |  |  |  |  | **0.021** |  | 6.090 | 0.944 – 39.279 | | 0.057 |
|  | Mucinous | | 11 |  |  |  |  |  |  |  |  |  |  | |  |

Abbreviations: CEA, carcinoembryonic antigen; CI, confidence interval; CRC, colorectal cancer; *GALNT14*, N-acetylgalactosaminyltransferase 14; HR, hazard ratio

Supplementary Table 4. Analysis of factors that Influenced recurrence free survival and overall survival using data of patients ≤ 65 years (n = 247)

|  | | | | **Recurrence Free Survival** | | | | |  | **Overall Survival** | | | | | |
| --- | --- | --- | --- | --- | --- | --- | --- | --- | --- | --- | --- | --- | --- | --- | --- |
|  | |  | | **Univariate Analysis** |  | **Multivariate Analysis** | | |  | **Univariate Analysis** |  | **Multivariate Analysis** | | | |
| **Parameters** | | **n** | | **P value** |  | **HR** | **95% CI** | **P value** |  | **P value** |  | **HR** | | **95% CI** | **P value** |
| Gender | Male | | 134 | 0.151 |  |  |  |  |  | 0.241 |  |  | |  |  |
|  | Female | | 113 |  |  |  |  |  |  |  |  |  | |  |  |
| CEA (ng/ml) | ≤ 5 | | 176 | 0.069 |  |  |  |  |  | 0.157 |  |  | |  |  |
|  | > 5 | | 71 |  |  |  |  |  |  |  |  |  | |  |  |
| GALNT14 “TT” | No | | 200 | 0.909 |  |  |  |  |  | **0.024** |  | 3.232 | 0.770 – 13.572 | | 0.109 |
|  | Yes | | 47 |  |  |  |  |  |  |  |  |  |  | |  |
| **Tumor status** |  | |  |  |  |  |  |  |  |  |  |  |  | |  |
| Location | Right | | 91 | 0.739 |  |  |  |  |  | 0.933 |  |  |  | |  |
|  | Left | | 156 |  |  |  |  |  |  |  |  |  |  | |  |
| Size (cm) | ≤4 | | 118 | **0.008** |  | 0.470 | 0.166 - 1.329 | 0.154 |  | 0.762 |  |  |  | |  |
|  | >4 | | 129 |  |  |  |  |  |  |  |  |  |  | |  |
| Surface area (cm^2^) | ≤14 | | 126 | **0.022** |  | 0.898 | 0.315 - 2.561 | 0.840 |  | 0.912 |  |  |  | |  |
|  | >14 | | 121 |  |  |  |  |  |  |  |  |  |  | |  |
| Free margin (cm) | ≤ 5.5 | | 128 | 0.611 |  |  |  |  |  | 0.519 |  |  |  | |  |
|  | > 5.5 | | 119 |  |  |  |  |  |  |  |  |  |  | |  |
| T stage | T1-3 | | 190 | 0.098 |  |  |  |  |  | **0.004** |  | 6.965 | 1.306 – 37.133 | | **0.023** |
|  | T4 | | 57 |  |  |  |  |  |  |  |  |  |  | |  |
| N stage | N1 | | 129 | **0.006** |  | 2.407 | 1.328 - 4.193 | **0.002** |  | 0.239 |  |  |  | |  |
|  | N2 | | 118 |  |  |  |  |  |  |  |  |  |  | |  |
| Differentiation | Well/moderate | | 212 | 0.827 |  |  |  |  |  | 0.311 |  |  |  | |  |
|  | Poor | | 35 |  |  |  |  |  |  |  |  |  |  | |  |
| Histology | Non-mucinous | | 225 | 0.978 |  |  |  |  |  | **0.028** |  | 3.220 | 0.746 – 13.906 | | 0.117 |
|  | Mucinous | | 22 |  |  |  |  |  |  |  |  |  |  | |  |

Abbreviations: CEA, carcinoembryonic antigen; CI, confidence interval; CRC, colorectal cancer; *GALNT14*, N-acetylgalactosaminyltransferase 14; HR, hazard ratio

Supplementary Table 5. Analysis of factors that Influenced recurrence free survival and overall survival using data of male patients (n = 167)

|  | | | | **Recurrence Free Survival** | | | | |  | **Overall Survival** | | | | | |
| --- | --- | --- | --- | --- | --- | --- | --- | --- | --- | --- | --- | --- | --- | --- | --- |
|  | |  | | **Univariate Analysis** |  | **Multivariate Analysis** | | |  | **Univariate Analysis** |  | **Multivariate Analysis** | | | |
| **Parameters** | | **n** | | **P value** |  | **HR** | **95% CI** | **P value** |  | **P value** |  | **HR** | | **95% CI** | **P value** |
| Ages (years) | ≤ 65 | | 134 | 0.213 |  |  |  |  |  | 0.456 |  |  | |  |  |
|  | > 65 | | 33 |  |  |  |  |  |  |  |  |  | |  |  |
| CEA (ng/ml) | ≤ 5 | | 115 | 0.259 |  |  |  |  |  | 0.331 |  |  | |  |  |
|  | > 5 | | 52 |  |  |  |  |  |  |  |  |  | |  |  |
| GALNT14 “TT” | No | | 136 | 0.614 |  |  |  |  |  | **0.030** |  | 6.147 | 1.006 – 37.565 | | **0.049** |
|  | Yes | | 31 |  |  |  |  |  |  |  |  |  |  | |  |
| **Tumor status** |  | |  |  |  |  |  |  |  |  |  |  |  | |  |
| Location | Right | | 65 | 0.177 |  |  |  |  |  | 0.802 |  |  |  | |  |
|  | Left | | 102 |  |  |  |  |  |  |  |  |  |  | |  |
| Size (cm) | ≤4 | | 80 | 0.073 |  |  |  |  |  | 0.314 |  |  |  | |  |
|  | >4 | | 87 |  |  |  |  |  |  |  |  |  |  | |  |
| Surface area (cm^2^) | ≤14 | | 80 | **0.041** |  | 0.516 | 0.273 - 0.972 | **0.041** |  | 0.322 |  |  |  | |  |
|  | >14 | | 87 |  |  |  |  |  |  |  |  |  |  | |  |
| Free margin (cm) | ≤ 5.5 | | 87 | 0.455 |  |  |  |  |  | 0.904 |  |  |  | |  |
|  | > 5.5 | | 80 |  |  |  |  |  |  |  |  |  |  | |  |
| T stage | T1-3 | | 137 | 0.614 |  |  |  |  |  | **0.009** |  | 8.571 | 1.552 – 47.326 | | **0.014** |
|  | T4 | | 30 |  |  |  |  |  |  |  |  |  |  | |  |
| N stage | N1 | | 90 | 0.174 |  |  |  |  |  | 0.274 |  |  |  | |  |
|  | N2 | | 77 |  |  |  |  |  |  |  |  |  |  | |  |
| Differentiation | Well/moderate | | 143 | 0.729 |  |  |  |  |  | 0.835 |  |  |  | |  |
|  | Poor | | 24 |  |  |  |  |  |  |  |  |  |  | |  |
| Histology | Non-mucinous | | 151 | 0.910 |  |  |  |  |  | 0.091 |  |  |  | |  |
|  | Mucinous | | 16 |  |  |  |  |  |  |  |  |  |  | |  |

Abbreviations: CEA, carcinoembryonic antigen; CI, confidence interval; CRC, colorectal cancer; *GALNT14*, N-acetylgalactosaminyltransferase 14; HR, hazard ratio

Supplementary Table 6. Analysis of factors that Influenced recurrence free survival and overall survival using data of left CRC patients (n = 187)

|  | | | | **Recurrence Free Survival** | | | | |  | **Overall Survival** | | | | | |
| --- | --- | --- | --- | --- | --- | --- | --- | --- | --- | --- | --- | --- | --- | --- | --- |
|  | |  | | **Univariate Analysis** |  | **Multivariate Analysis** | | |  | **Univariate Analysis** |  | **Multivariate Analysis** | | | |
| **Parameters** | | **n** | | **P value** |  | **HR** | **95% CI** | **P value** |  | **P value** |  | **HR** | | **95% CI** | **P value** |
| Ages (years) | ≤ 65 | | 156 | 0.729 |  |  |  |  |  | 0.548 |  |  | |  |  |
|  | > 65 | | 31 |  |  |  |  |  |  |  |  |  | |  |  |
| Gender | Male | | 102 | 0.098 |  |  |  |  |  | 0.250 |  |  | |  |  |
|  | Female | | 85 |  |  |  |  |  |  |  |  |  | |  |  |
| CEA (ng/ml) | ≤ 5 | | 130 | **0.010** |  | 2.221 | 1.208 - 4.081 | **0.010** |  | 0.164 |  |  | |  |  |
|  | > 5 | | 57 |  |  |  |  |  |  |  |  |  | |  |  |
| GALNT14 “TT” | No | | 154 | 0.301 |  |  |  |  |  | **0.026** |  | 1.131 | 0.763 – 13.918 | | 0.922 |
|  | Yes | | 33 |  |  |  |  |  |  |  |  |  |  | |  |
| **Tumor status** |  | |  |  |  |  |  |  |  |  |  |  |  | |  |
| Size (cm) | ≤4 | | 103 | 0.178 |  |  |  |  |  | 0.274 |  |  |  | |  |
|  | >4 | | 84 |  |  |  |  |  |  |  |  |  |  | |  |
| Surface area (cm^2^) | ≤14 | | 111 | 0.341 |  |  |  |  |  | 0.356 |  |  |  | |  |
|  | >14 | | 76 |  |  |  |  |  |  |  |  |  |  | |  |
| Free margin (cm) | ≤ 5.5 | | 130 | 0.157 |  |  |  |  |  | 0.754 |  |  |  | |  |
|  | > 5.5 | | 57 |  |  |  |  |  |  |  |  |  |  | |  |
| T stage | T1-3 | | 150 | 0.700 |  |  |  |  |  | **0.007** |  | 23.298 | 1.528 – 355.153 | | **0.024** |
|  | T4 | | 37 |  |  |  |  |  |  |  |  |  |  | |  |
| N stage | N1 | | 88 | 0.174 |  |  |  |  |  | 0.204 |  |  |  | |  |
|  | N2 | | 99 |  |  |  |  |  |  |  |  |  |  | |  |
| Differentiation | Well/moderate | | 172 | 0.514 |  |  |  |  |  | **0.004** |  | 6.020 | 0.104 - 349.300 | | 0.386 |
|  | Poor | | 15 |  |  |  |  |  |  |  |  |  |  | |  |
| Histology | Non-mucinous | | 177 | 0.541 |  |  |  |  |  | **0.003** |  | 5.772 | 0.122 - 272.140 | | 0.373 |
|  | Mucinous | | 10 |  |  |  |  |  |  |  |  |  |  | |  |

Abbreviations: CEA, carcinoembryonic antigen; CI, confidence interval; CRC, colorectal cancer; *GALNT14*, N-acetylgalactosaminyltransferase 14; HR, hazard ratio
